# Quaternary Ammonium Biocide Resistance in Non-Typhoidal *Salmonella* from Pig Carcasses

**DOI:** 10.3390/vetsci13030247

**Published:** 2026-03-05

**Authors:** Lorina Lourenço, Vanessa Ferreira da Silva, Madalena Vieira-Pinto, Manuela Oliveira, João B. Cota

**Affiliations:** 1 CIISA—Centre for Interdisciplinary Research in Animal Health, Faculty of Veterinary Medicine, University of Lisbon, Avenida da Universidade Técnica, 1300-477 Lisbon, Portugalmoliveira@fmv.ulisboa.pt (M.O.); 2 Veterinary and Animal Research Centre (CECAV), University of Trás-os-Montes and Alto Douro (UTAD), 5000-801 Vila Real, Portugalmmvpinto@utad.pt (M.V.-P.); 3 Associate Laboratory for Animal and Veterinary Science (Al4AnimalS), Av. da Universidade Técnica, 1300-477 Lisbon, Portugal; 4 cE3c—Centre for Ecology, Evolution and Environmental Changes & CHANGE—Global Change and Sustainability Institute, Faculty of Sciences, University of Lisbon, Campo Grande, 1749-016 Lisbon, Portugal

**Keywords:** *Salmonella*, QAC, resistance, resistance genes, pig, pork

## Abstract

Non-typhoidal *Salmonella* (NTS) are one of the most common foodborne pathogens worldwide, and pork is a major food vehicle together with eggs and poultry meat. Contamination of pork within food processing facilities, such as slaughterhouses, can be associated with persistence of *Salmonella* in the environment due to biocide resistance. In this study, we assessed the susceptibility of NTS isolates from pig carcasses to a QAC-based commercial formulation according to the EN 1656/2009 standard and the presence of QAC resistance genes through PCR. The *qacEΔ1* and *qacF* genes were found in 31.8% and 29.5% of the isolates respectively, while *qacE* was absent. All isolates were found to be susceptible at a tested concentration 10 times lower (0.1%) than the minimum in-use recommended concentration, with MIC values below 0.1% (≈70 mg/L of Benzalkonium Chloride). Our findings point towards the importance of correct cleaning and disinfection protocols and the role of good hygiene practices as corrective and/or preventive measures to avoid cross-contamination.

## Simple Summary

In this study, we assessed the resistance ability of *Salmonella* isolated from pig carcasses to a commercial quaternary ammonium compound (QAC) disinfectant formulation and examined the prevalence of genes that are associated with such resistance and with possible persistence in the contaminated surfaces. Our results show that though the isolates carried resistance genes, they were susceptible to the disinfectant formulation, even at concentrations 10 times lower than the minimum recommended in-use concentration. These results show that the possible persistence of these bacteria in the environment of food processing facilities, such as slaughterhouses, should not be exclusively associated with disinfectant resistance, stressing the importance of correct cleaning and disinfection procedures and/or hygiene practices.

## 1. Introduction

Non-typhoidal *Salmonella* (NTS) are still major foodborne pathogens despite the efforts to limit their impact on human health. It is estimated that between 1.2 and 1.35 million individuals fall ill with salmonellosis each year in the United States of America [1,2] and that NTS is responsible for 12,500 hospitalizations [3]. These numbers are only surpassed by Norovirus and *Campylobacter* spp., being estimated to cause 5.5 million and 1.9 million cases of disease/year, respectively [3]. In the European Union, during 2024, *Salmonella* was, alike the previous years, the second most commonly reported zoonotic pathogen, after *Campylobacter* spp., being responsible for 79,703 cases of illness, and the most frequently reported causative agent of multi-country foodborne outbreaks [4]. Though not exclusively, the major sources of human salmonellosis are animal-derived foods, namely eggs, poultry meat, pork and beef [5,6], especially in high-income countries where diarrheal NTS infections are classified as a foodborne disease [7]. Indeed, food-producing animals are the most relevant reservoirs of the multiple NTS serotypes responsible for human illness cases [8], and an association of some of the most relevant serotypes with specific animal species and/or animal-derived foods is frequently observed in foodborne outbreaks [9,10,11]. Regarding the isolation of *Salmonella* in pigs and along the pork production chain in Europe, the most frequently reported serotypes are Typhimurium, Derby, Rissen and the monophasic variant of Typhimurium [12].

Concerning meat and thereof products, contamination can take place along the multiple steps of the production chain, starting at abattoirs, mostly arising from the evisceration of *Salmonella* asymptomatic carriers and the contamination of the abattoir environment and equipment, up to processing and distribution, through cross-contamination and manipulation [13]. This scenario is also true for the pork production chain, with pork and pork products being among the food categories consistently implicated in salmonellosis foodborne outbreaks [14,15,16]. Besides the obvious consequences for human health of *Salmonella*-contaminated pork, the associated economic impacts should not be neglected, making it a multidimensional problem. It was previously estimated that the annual economic burden of foodborne illness of pork-associated *Salmonella* in the United States of America was 1.9 billion US dollars, ranking this food–pathogen pair as one of the most costly meat-related foodborne illnesses [17]. Additionally, the economic consequences of surveillance and control strategies at different levels are also relevant [18,19,20,21], as well as the economic costs of possible market recalls and limited market access [22].

One of the possible roots of cross-contamination of meat is the persistence of *Salmonella* in abattoirs and meat processing plants, where persistence of multiple *Salmonella* serotypes has been reported [23]. As reviewed previously [24], persistence refers to the ability of microorganisms to survive for extended periods of time in certain habitats; specifically in food processing environments, it means that the same bacterial strain is repeatedly isolated from the same location for months or years. It seems that the persistence of *Salmonella* in such environments results from a combination of different features, including the ability to form biofilms, presence and expression of virulence genes and ability to resist to antimicrobials and/or to disinfectants [23]. On the other hand, contamination arising from the environment and equipment can occur simply due to the inadequate application of cleaning and disinfection protocols, since it is generally believed that proper disinfection reduces the risk of *Salmonella* cross-contamination [25].

The correct use of biocides for disinfection purposes is essential to eliminate and/or prevent the growth of unwanted microorganisms in the surfaces and environments of diverse settings, namely those associated with food production, where such chemical substances are widely used [26]. Quaternary ammonium compounds (QACs) are frequently applied in these settings, belonging to the group of nonoxidizing antimicrobial biocides and being available in different formulations for domestic, industrial and healthcare usage. The antimicrobial activity of QACs is mainly associated with their ability to interact with the cytoplasmic membrane of microorganisms, leading to leakage of intracellular material, but they can also affect intracellular targets, degrading proteins and DNA [27,28]. There is an increasing concern regarding the loss of effectiveness of biocides through the development of bacterial resistance, and to the possibility of cross-resistance with other unrelated antimicrobial compounds [29]. Intrinsic resistance to QACs is mostly associated with membrane permeability barriers and with the activity of efflux pumps, while acquired resistance may result from either the overexpression of the chromosome-encoded efflux pumps, the reduction in membrane permeability, or the acquisition of efflux pump genes encoded in mobile genetic elements [30]. Among the genes associated with QAC resistance, the presence of *qac* genes have been studied in both Gram-positive and Gram-negative bacteria [31]. These genes, including *qacE*, *qacF*, *qacH* and *qacI*, among others, are carried in integrons, transposons, plasmids and other mobile genetic elements [32]. Additionally, *qacEΔ1*, considered to be a variant of *qacE*, is commonly found in Gram-negative bacteria, including in *Enterobacteriaceae* [31].

The aim of this study was to investigate the susceptibility of a collection of NTS isolated from pig carcasses to a commercial QAC formulation, under in vitro conditions, along with the presence of QAC resistance-associated genes.

## 2. Materials and Methods

### 2.1. Bacterial Isolates

A collection of 44 *Salmonella* spp. isolates originating from pigs slaughtered in a Portuguese abattoir located in the northern region of the country, previously characterized [33], was used in this study. Briefly, the isolation of *Salmonella* from skin after stunning and bleeding procedures, and from the external and internal carcass surfaces before cooling was achieved according to the ISO 6579:2002 standard [34]. All presumptive *Salmonella* isolates were serotyped according to the Kauffman-White scheme at the Instituto Nacional de Investigação Agrária e Veterinária (INIAV), the Portuguese National Reference Laboratory for *Salmonella* identification in animal and food samples. Additionally, the bacterial isolates were subjected to genomic fingerprinting by Pulse Field Gel Eletrophoresis (PFGE) using the PulseNet protocol [35], and the PFGE patterns were studied by computer-assisted cluster analysis using BioNumerics^®^ version 6.6 software (Applied Maths, SintMartens-Lantem, Belgium).

### 2.2. Detection of Biocide Resistance Genes

PCR amplification was conducted to detect genes for QAC resistance genes. After bacterial DNA extraction performed according to the protocol of Moore et al. (2004) [36], the amplification of *qacEΔ1*, *qacE* and *qacF* gene was performed using previously reported primers [37]. Each PCR reaction was performed in a volume of 25 μL and consisting of 12.5 μL of NZYTaq II 2x Green Master Mix (NZYtech^®^, Lisbon, Portugal), 9.5 μL of nuclease-free water, 1 μL of forward primer (STAB VIDA, Lda., Caparica, Portugal) at a final concentration of 0.4 μM, 1 μL of reverse primer (STAB VIDA, Lda., Caparica, Portugal) at a final concentration of 0.4 μM and 1 μL of DNA from each isolate. The presence or absence of amplicons was confirmed by gel electrophoresis, in a 2% agarose gel stained with GreenSafe Premium^®^ (NZYtech^®^, Lisbon, Portugal), and visualized using ChemiDoc XRS+ (BIO-RAD Laboratories, Inc., Algés, Portugal).

### 2.3. Determination of Minimum Inhibitory Concentration (MIC) and Minimum Bactericidal Concentration (MBC)

Twelve isolates harboring different biocide resistance gene combinations from the different *Salmonella* serotypes, from the different sampled regions (skin, and external and internal carcass surfaces) and from the different genomic clusters were selected for further characterization. *Enterococcus hirae* ATCC 10541, *Staphylococcus aureus* ATCC 6538, *Escherichia coli* ATCC 10536 and *Pseudomonas aeruginosa* ATCC 15442 were used as control strains.

The MIC and MBC of Suma Bac D10^®^ (Diversey™), a QAC-based sanitizer formulation widely used in food handling and processing units, with Benzalkonium Chloride (BKC) as the biocide active substance at the concentration of 70 g/kg, was determined according to the EN 1656:2009 standard for the evaluation of bactericidal activity of chemical disinfectants and antiseptics used in the veterinary field [38]. Considering that the recommended manufacturer’s in-use concentrations vary between 1% (≈700 mg/L) and 4% (≈2800 mg/L), the commercial biocide formulation was diluted in sterile water to obtain the following eight concentrations to be tested—5% (≈3500 mg/L), 4% (≈2800 mg/L), 3% (≈2100 mg/L), 2% (≈1400 mg/L), 1% (≈700 mg/L), 0.5% (≈350 mg/L), 0.25% (≈125 mg/L), and 0.1% (≈70 mg/L). For neutralization of the biocide activity, a solution containing 30 g/L polysorbate 80 (Merck & Co., Inc., Rahway, NJ, USA), 30 g/L saponin (SigmaAldrich, St. Louis, MO, USA) and 3 g/L ovolecithin (The British Drug Houses Ltd., London, UK) was used [38]. Additionally, to simulate the presence of organic matter, the experiments were executed using low and high doses of interfering substances [38]. Thus, the MIC and MBC of the biocide were determined in three different conditions, namely with no interfering substances, and both with low and high levels of interfering substances. The solution of low-level interfering substance (LIS) was prepared by dissolving 3 g of bovine albumin fraction V (NZYTech^®^, Lisbon, Portugal) in 100 mL of water, followed by sterilization by membrane filtration with a 0.2 µm syringe filter (Nalgene^®^, New York, NY, USA). The high-level interfering substance (HIS) solution was prepared using two solutions. The first one was prepared by dissolving 50 g of yeast extract (Oxoid, Ltd., Hampshire, UK) in 250 mL of water, followed by sterilization by autoclave (120 °C, 20 min). The second one was prepared by dissolving 5 g of albumin in 25 mL of water, followed by sterilization with a 0.2 µm syringe filter (Nalgene^®^, New York, NY, USA). To obtain the final HIS solution, 25 mL of the yeast extract suspension were added to this last solution.

All experiments were carried out in 96-well plates (VWR International^®^, Leuven, Belgium). Briefly, using 24 h bacterial cultures grown on Brain Heart Infusion agar (VWR International^®^, Leuven, Belgium), bacterial suspensions of the selected isolates were prepared in sterile saline solution at a concentration corresponding to 0.5 McFarland (~1.5 × 10^8^ CFU/mL). Then, the assay was performed using three microplates. In the first 96-well plate, 20 μL of the bacterial suspensions were added to pre-filled wells containing 160 μL of the biocide formulation at the different concentrations previously mentioned and either 20 μL of sterile water of LIS or of HIS. The mixture was incubated with agitation (700 rpm) for 5 min ± 10 s, as per the disinfectant manufacturer’s instructions. After, 20 μL of the mixture of each well of the first plate were transferred to the corresponding well of a second plate already containing 160 μL of the neutralizing solution and 20 μL of sterile water. The plates were incubated with agitation (700 rpm) for 5 min ± 10 s [38]. After neutralization, 20 μL of each well were added to the corresponding wells of a third plate, pre-filled with 180 μL of Tryptone Soya Broth (TSB) (Oxoid, Ltd., Hampshire, UK). Moreover, this third plate included both negative (wells with 200 μL of non-inoculated TSB) and positive controls (wells pre-filled with 180 μL of the bacterial suspensions in TSB). This last plate was incubated for 24 h at 37 °C, after which the MIC was visually determined as the minimum concentration of the biocide formulation able to inhibit bacterial multiplication.

To determine the MBC, 5 μL were taken from wells where no bacterial multiplication was observed, followed by inoculation on Tryptone Soya Agar (TSA) (Oxoid, Ltd., Hampshire, UK) and incubation for 24 h at 37 °C.

To evaluate the reproducibility of the experiments, the procedures were repeated for 10% of the 12 isolates tested, which were randomly selected.

### 2.4. Efflux Pump Activity Assay

Efflux pump activity was assessed using the ethidium bromide (EtBr) agar cartwheel method [39]. Briefly, the agar plates were prepared using Luria Broth agar (NZYTech^®^, Lisbon, Portugal) and EtBr. EtBr was prepared from a stock solution diluted to obtain the desired final concentration and added to the LB agar after sterilization. Assays were performed using five different EtBr concentrations, namely 0.50, 1.00, 1.50, 2.00 and 2.50. The plates were inoculated by swabbing, with freshly prepared 0.5 McFarland bacterial suspensions of the 12 selected isolates. The plates were then incubated for 18 h at 37 °C, protected from light. After incubation, the presence or absence of fluorescence was assessed by UV transillumination using ChemiDoc XRS+ (BIO-RAD Laboratories, Inc., Algés, Portugal). In each assay, *Enterococcus faecium* CCUG 36804, with high efflux pump activity, and *Enterococcus faecalis* ATCC 29212, with no efflux pump activity, were also tested.

### 2.5. Statistical Analysis

The possible statistical associations between the presence of QAC resistance gene and isolate characteristics were evaluated using Fisher’s exact test performed on IBM SPSS Statistics version 28 (SPSS Inc. Chicago, IL, USA). Statistically significant differences corresponded to *p* values lower than 0.05.

## 3. Results

### 3.1. Detection of QAC Resistance Genes

None of the isolates harbored the *qacE* gene (Table 1). The presence of *qacEΔ1* was observed in 31.8% (14/44) of isolates and *qacF* was found in 29.5% (13/44) of isolates. Thus, regarding QAC resistance genotypes, the most frequent were *qacEΔ1* present in 15.9% (7/44), *qacEΔ1/qacF* in 15.9% (7/44) followed by *qacF* in 13.6% (6/44).

In the collection under study, the frequency of *qacEΔ1* was diverse between serotypes, being present in 23.8% (5/21) of the *Salmonella* 4,[5],12:i:- isolates, in 22.2% (4/18) of the Rissen isolates and in all five Derby isolates (*p* < 0.05). On the other hand, *qacF* was mostly found in Rissen isolates, with a frequency of 55.6% (10/18), being less frequent in *Salmonella* 4,[5],12:i:- isolates (14.3%; 3/21) and absent in the Derby isolates (*p* < 0.05).

Focusing on the genomic clusters, the presence of those resistance genes was more common in some clusters when compared to others, as observed for *qacF* in cluster IB (90%) and for *qacEΔ1* in clusters III (100%) and IB (40%) (*p* < 0.05).

### 3.2. Minimum Inhibitory Concentration and Minimum Bactericidal Concentration

The mean Suma Bac D10^®^ MIC and MBC values found for the 12 selected isolates is presented in Table 2.

In all assays, including those performed without interfering substance (NIS), with low interfering substance (LIS) or with high interfering substance (HIS), the MIC values obtained were below the lowest recommended concentration for the biocide formulation studied (0.1%), since in all three conditions (NIS, LIS and HIS), the lowest concentration tested (0.1%) had a bactericidal effect (MBC) for all the isolates studied. No differences between MIC or MBC values and the diverse QAC resistance genotypes, genomic clusters or serotypes were observed.

### 3.3. Efflux Pump Activity

The level of efflux activity of the previously 12 selected isolates was evaluated using the EtBr cartwheel method. After incubation, none of the isolates showed any efflux activity at any of the EtBr concentrations tested, showing a similar level of fluorescence as *Enterococcus faecalis* ATCC 29212 (negative for efflux activity included in each test).

## 4. Discussion

In this study, the susceptibility of a collection of *Salmonella* isolates collected from pig carcasses to a QAC commercial biocide formulation was assessed in in vitro conditions, in addition to the presence of QAC resistance genes (*qacE*, *qacEΔ1* and *qacF*). This research was designed to better understand the possible persistence ability of *Salmonella* isolates of the pork production chain in food processing environments and the association with their resistance ability to a biocidal active substance commonly used in the agri-food industry.

Firstly, all 44 isolates were screened for the presence of QAC resistance genes through conventional PCR. The presence of specific genes coding for efflux pumps has been pointed out as one of the possible mechanisms for QAC resistance. Such genes, namely *qacE*, *qacF*, *qacG*, *qacH* and *qacI*, have been associated with reduced susceptibility to QACs in Gram-negative bacteria [30]. Additionally, *qacEΔ1* gene, a variation of the *qacE* gene, has been found to be present in Gram-negative bacteria [40], including in different *Salmonella* serotypes [41]. Furthermore, these genes can be carried together in mobile genetic elements, along with other antimicrobial resistant determinants [42]. Our results show that almost a third (31.8%) of all isolates harbored the *qacEΔ1* gene, isolated or in combination with *qacF*, while *qacE* was not detected. The prevalence of such biocide resistance genes in *Salmonella* isolated from pigs in Portugal is still a knowledge gap, with limited information available. Nevertheless, our results are in accordance with a previous report focusing on *Salmonella* isolates from pig and poultry, obtained from feces, rectal swabs, drinking water and feed samples, which described a *qacEΔ1* prevalence of 27% and the absence of *qacE* [43]. The *qacEΔ1* gene is typically associated with class 1 integrons resulting from a deletion of *qacE* gene, found fused with the sulfonamide resistance gene *sul1* [44]. Previously, these integrons have been found in 14 *Salmonella* Rissen and 15 *Salmonella* Typhimurium isolates belonging to a collection of 40 antibiotic resistant *Salmonella* isolates from fecal samples of domestic pigs and wild boars in Portugal [45]. Our results revealed that the presence of *qacEΔ1* was associated with the Derby serotype, with all of the isolates belonging to that serotype harboring the QAC resistance gene.

Besides *qacEΔ1*, *qacF* was also found in 29.5% of the isolates, alone or in combination with *qacEΔ1* gene, mostly among *Salmonella* Rissen isolates. Both *qacE* and *qacF* show a high degree of similarity, being found in class 1 integrons [46], though the prevalence of the latter has not been a subject of extensive research, being considered to occur sporadically [31]. The presence of *qacEΔ1* and *qacF* simultaneously was observed in 15.9% of the isolates, from both the Rissen and 4,[5],12:i:- serotypes. These biocide resistance genes have been found in *Salmonella* from other settings, namely from poultry and egg production chains [47,48] and retail food of animal origin [49], but similar data focusing on pig and/or pork is scarce. Further studies are needed to achieve a clearer assessment of the actual dissemination of these and other QAC resistance genes, along with the sequence of class 1 integrons among *Salmonella* from the pork production chain.

From the 44 isolates studied, 12 were selected for biocide susceptibility testing according to the EN 1656:2009 protocol. In total, eight concentrations of QAC commercial formulation were tested, considering the manufacturer’s recommendations. After validation of the protocol, all isolates were found to be susceptible to every biocide concentration tested, since the MBC obtained was the lowest concentration tested (0.1% ≈ 70 mg/L of BKC), regardless of the absence or presence of organic matter. This concentration should be interpreted as the lowest attaining a bactericidal effect in our experimental conditions and not as the actual MBC of the QAC formulation towards the tested *Salmonella* isolates. In fact, the MBC of BKC for *Salmonella* can be as low as 0.003 mg/L [50], which is about 20,000-fold lower than the lowest concentration tested in our study. As previously reviewed [51], the MIC of BKC for *Salmonella* isolates is quite variable, ranging from concentrations as low as 0.003 mg/L to 256 mg/L. In the present work, under the conditions tested, it was not possible to calculate the actual MIC for any of the isolates and the presented result, <0.1%, should be carefully interpreted.

Though the presence of *qacEΔ1* gene was not previously associated with increased BKC MIC values in *Salmonella* isolates from poultry and swine [43], a more recent study has found that class 1 integrons carrying that gene conferred resistance to both antibiotics and disinfectants in *Salmonella*, namely to BKC [52]. The influence of the presence of *qacEΔ1* and *qacF* genes on the MIC and MBC values could not be attained in our study, as this could only be achieved after the determining the actual MIC and MBC values towards isolates with and without these genes. This approach could also be improved by expanding the number of isolates tested in order to grasp the possible variability that was not detected in the present experimental design. Determining the actual influence of disinfectant resistance genes on the *Salmonella* isolates, in light of the findings of the present study, is important for a better understanding of their impact on phenotypic biocide susceptibility and should therefore be addressed in future research. Nevertheless, all results should also be interpreted through the practical perspective, considering the discrepancy between the BKC concentrations tested in vitro and those in-use.

The QAC resistance genes studied encode for transmembrane efflux pumps, whose expression, in theory, should provide bacteria with increased resistance to QAC biocides. Thus, the level of efflux pump activity was assessed through a semi-quantitative method, exposing the isolates to different concentrations of EtBr. A similar approach was previously pursued to study the level of efflux pump activity of multi-drug resistant NTS from food-producing animals and handlers [53]. It was not possible to observe any increase in efflux pump activity when comparing isolates harboring or not harboring the QAC resistance genes and the negative control. Whether these genes are in fact silent or being expressed at low levels should be assessed through finer molecular methodologies in future studies.

Above all, even when increased MIC and MBC values of BKC were reported, the bacterial strains tested were still susceptible to the in-use concentrations [51]. In our study, and despite the presence of QAC resistance genes, the studied isolates were susceptible to the QAC formulation, even at a 10-fold dilution of the lowest in-use concentration. This result is in line with the fact that commercial biocide formulations are established so that the in-use concentration is more than 1000-fold higher than the biocides’ MIC [51]. The concentration of the biocide is one of the factors that can contribute to the development of resistance or reduced susceptibility in bacteria, namely through the application of incorrect dilutions of active substances or formulations [54]. Though such improper dilutions are not likely to be applied in animal husbandry and/or food processing environments, bacteria in natural environments can be exposed to sub-inhibitory concentrations of biocides, as the substances used in the cleaning and disinfection procedures can be washed away and reach waste waters [55]. For *Salmonella*, it has been shown that exposure to sub-inhibitory concentrations of BKC can lead to increased MIC and MBC values [50,56,57].

Overall, our results highlight the importance of the correct and judicious use of antimicrobial biocides, and the implementation of proper cleaning and disinfection protocols and good hygiene practices in food processing environments, especially in abattoirs, to effectively control *Salmonella* cross-contamination.

## 5. Conclusions

The present study reports the susceptibility of NTS isolates from pig carcasses to a QAC-based commercial formulation. The QAC resistance genes *qacEΔ1* and *qacF* were identified among the isolates studied; however, their impact on the susceptibility to the biocide formulation was not fully evaluated under the experimental conditions used, thus further studies should be undertaken to better understand their role. All isolates tested were susceptible to the QAC formulation at concentrations 10 times lower (≈70 mg/L of BKC) than the minimum recommended in-use concentration. The present work brings new insights within the framework of *Salmonella* control in the pork production chain.

## Figures and Tables

**Table 1:** Detection of the QAC resistance genes *qacEΔ1*, *qacE* and *qacF* by PCR amplification in the *Salmonella* isolates under study and corresponding resistance genotype.

Isolate	*qacEΔ1*	*qacE*	*qacF*	Genotype	Cluster	Serotype
p1	−	−	−	−	IA	Rissen
p3	−	−	−	−	IA	
p4	−	−	−	−	IA	
p5	−	−	−	−	IA	
p58	−	−	−	−	IA	
p116	−	−	−	−	IA	
ci57	−	−	−	−	IA	
p25	−	−	+	*qacF*	IB	Rissen
p31	−	−	+	*qacF*	IB	
p61	−	−	+	*qacF*	IB	
p62	−	−	+	*qacF*	IB	
p64	+	−	+	*qacEΔ1/qacF*	IB	
ce21	+	−	+	*qacEΔ1/qacF*	IB	
ce37	+	−	+	*qacEΔ1/qacF*	IB	
ce44	−	−	+	*qacF*	IB	
ci21	+	−	+	*qacEΔ1/qacF*	IB	
ci55	−	−	−	−	IB	
p55	+	−	-	*qacEΔ1*	IIA	4,[5],12:i:-
p56	−	−	−	−	IIA	
p96	−	−	−	−	IIB	4,[5],12:i:-
p104	−	−	−	−	IIB	
p106	+	−	+	*qacEΔ1/qacF*	IIB	
p107	−	−	−	−	IIB	
p109	+	−	+	*qacEΔ1/qacF*	IIB	
p110	−	−	−	−	IIB	
p112	−	−	−	−	IIB	
p114	−	−	−	−	IIB	
p115	−	−	−	−	IIB	
p118	−	−	−	−	IIB	
ci104	+	−	+	*qacEΔ1/qacF*	IIB	
ci105	−	−	−	−	IIB	
ci108	−	−	−	−	IIB	
ci109	−	−	−	−	IIB	
ci110	−	−	−	−	IIB	
ci111	−	−	−	−	IIB	
ci115	−	−	−	−	IIB	
ci116	−	−	−	−	IIB	
ci117	+	−	−	*qacEΔ1*	IIB	
p66	+	−	−	*qacEΔ1*	III	Derby
p67	+	−	−	*qacEΔ1*	III	
p68	+	−	−	*qacEΔ1*	III	
ce70	+	−	−	*qacEΔ1*	III	
ci68	+	−	−	*qacEΔ1*	III	
ci38	−	−	+	*qacF*	ind	Rissen

“p”—isolates from pig skin before scalding; “ci”—isolates from the internal surface of the carcass; “ce”—isolates that came from the external surface of the carcass; “+”—positive; “−”—negative; IA and IB—genomic clusters associated with Rissen serotype; IIA and IIB—genomic clusters associated with Typhimurium from monophasic variant (4,[5],12:i:-); III—genomic cluster associated with Derby serotype; ind—independent genomic cluster [33].

**Table 2:** Suma Bac D10^®^ MIC and MBC medium values for the selected isolates under different testing conditions—no interfering substance (NIS), low interfering substance (LIS) and high interfering substance (HIS).

Isolate	Serotype	QAC Resistance Genotype	Cluster		MIC (%)			MBC (%)	
				NIS	LIS	HIS	NIS	LIS	HIS
p1	Rissen	−	IA	<0.100	<0.100	<0.100	0.100	0.100	0.100
ce21	Rissen	*qacEΔ1/qacF*	IB	<0.100	<0.100	<0.100	0.100	0.100	0.100
ce37	Rissen	*qacEΔ1/qacF*	IB	<0.100	<0.100	<0.100	0.100	0.100	0.100
ci21	Rissen	*qacEΔ1/qacF*	IB	<0.100	<0.100	<0.100	0.100	0.100	0.100
p64	Rissen	*qacEΔ1/qacF*	IB	<0.100	<0.100	<0.100	0.100	0.100	0.100
p55	4,[5],12:i:-	*qacEΔ1*	IIA	<0.100	<0.100	<0.100	0.100	0.100	0.100
p56	4,[5],12:i:-	−	IIA	<0.100	<0.100	<0.100	0.100	0.100	0.100
ci104	4,[5],12:i:-	*qacEΔ1/qacF*	IIB	<0.100	<0.100	<0.100	0.100	0.100	0.100
ci117	4,[5],12:i:-	*qacEΔ1*	IIB	<0.100	<0.100	<0.100	0.100	0.100	0.100
p109	4,[5],12:i:-	*qacEΔ1/qacF*	IIB	<0.100	<0.100	<0.100	0.100	0.100	0.100
ce70	Derby	*qacEΔ1*	III	<0.100	<0.100	<0.100	0.100	0.100	0.100
ci38	Rissen	*qacF*	ind	<0.100	<0.100	<0.100	0.100	0.100	0.100
x^−^	−	−	−	0.100	0.100	0.100	0.100	0.100	0.100
^σ^	−	−	−	0.000	0.000	0.000	0.000	0.000	0.000

NIS—no interfering substance; LIS—low interfering substance; HIS—high interfering substance; x^−^—mean; ^σ^—standard deviation. “p”—isolates from pig skin before scalding; “ci”—isolates from the internal surface of the carcass; “ce”—isolates that came from the external surface of the carcass; “−”—negative; IA and IB—genomic clusters associated with Rissen serotype; IIA and IIB—genomic clusters associated with Typhimurium from monophasic variant (4,[5],12:i:-); III—genomic cluster associated Derby serotype; ind—independent genomic cluster [33].

## Data Availability

The original contributions presented in this study are included in the article. Further inquiries can be directed to the corresponding author.

## Abbreviations

The following abbreviations are used in this manuscript:

PCR	Polymerase Chain Reaction
